# Phytochemical composition and synergistic antimicrobial effects of *Rosmarinus officinalis* essential oils during flowering in an arid mediterranean region

**DOI:** 10.3934/microbiol.2025033

**Published:** 2025-10-30

**Authors:** Imane Abbad, Bouchra Soulaimani, Imane El Hakioui, Soraia El Baz, Elena Maria Varoni, Marcello Iriti

**Affiliations:** 1 Laboratory of Water Sciences, Microbial Biotechnologies, and Natural Resources Sustainability (AQUABIOTECH), Unit of Microbial Biotechnologies, Agrosciences, and Environment (BIOMAGE)-CNRST Labeled Research Unit No.4, Faculty of Sciences-Semlalia, University Cadi Ayyad, Marrakech, Morocco; 2 Agrosciences Program, College of Agriculture and Environmental Science, Mohammed VI Polytechnic, Ben Guerir 43150, Morocco; 3 Department of Biomedical, Surgical and Dental Sciences, University of Milan, Milan, Italy

**Keywords:** *Rosmarinus officinalis*, flowering period, essential oil, chemical composition, autumn period, antimicrobial activity, synergistic effects

## Abstract

*Rosmarinus officinalis* has attracted significant attention due to its broad-spectrum antimicrobial activity, largely attributed to its bioactive essential oils (EOs). Several studies indicate that the flowering period is crucial for harvesting rosemary's aerial parts for optimal EO extraction. However, its prolonged flowering period complicates the determination of an optimal harvest time, potentially affecting yield, chemical composition, and efficacy. This study provides, for the first time, a systematic month-by-month evaluation of EO yield, chemical composition, and synergistic antimicrobial potential of rosemary cultivated under arid Mediterranean conditions during its flowering period (September to March). EO samples were analyzed by GC-MS and assessed for antimicrobial activity against clinically relevant pathogens, including *S. aureus*, *E. coli*, *S. enterica*, and four *Candida* species. The synergistic potential was further evaluated with two conventional antimicrobials (streptomycin and amphotericin B). The results showed that the EO yields ranged from 1.73% to 2.75%, with a clear peak in autumn. GC-MS analysis identified 31 compounds, dominated by 1,8-cineole (27.57 ± 0.76%–36.28 ± 0.26%), α-pinene (15.36 ± 0.23%–28.97 ± 0.10%), and camphor (7.12 ± 0.00%–15.37 ± 0.12%), confirming the prevalence of the 1,8-cineole/α-pinene/camphor chemotype. Antimicrobial assays demonstrated stronger activity against fungal strains, particularly *C. krusei* and *C. albicans*, with enhanced efficacy observed in EOs collected in October-November. Synergy assays showed significant potentiation of streptomycin activity, particularly against *E. coli* in autumn, with up to a 32-fold increase in efficacy. In contrast, only limited synergistic effects were observed with amphotericin B. Overall, our findings emphasize the clinical relevance of optimizing harvest timing, as autumn-harvested rosemary EOs exhibit the greatest potential as natural antibiotic adjuvants against multidrug-resistant pathogens. Nevertheless, the partial antagonism with amphotericin B highlights the need for strain-specific compatibility assessments to avoid compromising drug efficacy in combinatory therapies.

## 1. Introduction

In recent years, the use of herbal medicine has seen significant growth in combating pathogenic bacteria, driven by the increasing emergence of bacterial resistance to existing antibiotics. This rise in multidrug-resistant infections has been linked to an alarming escalation in mortality rates annually. It is projected that if no measures are taken, antimicrobial resistance, currently causing around 700,000 deaths globally each year, could result in as many as 10 million deaths annually by 2050 [1],[2]. This urgent challenge has intensified the search for innovative treatments capable of combating multi-drug-resistant bacteria. Plant-derived essential oils (EOs) have emerged as a promising alternative due to their potent antimicrobial properties against resistant strains, their availability, and favorable perceptions among consumers [3]–[5]. Research has shown that various EOs from plants exhibit strong antimicrobial effects on both Gram-positive and Gram-negative bacteria, which is largely attributed to their complex chemical structures and the synergistic actions of their components [5]–[7].

*Rosmarinus officinalis* (rosemary), belonging to the Lamiaceae family, is one of the medicinal and aromatic plants that has attracted significant research interest. In fact, the species is known to be a rich source of EOs, which find extensive use across agri-food and pharmaceutical industries [8]. Its EOs are highly valued for their diverse biological and pharmacological properties, including antioxidant, antibacterial, and antifungal activities [9]. These biological properties have been attributed mainly to their richness in several bioactive molecules, like 1,8-cineole, borneol, β- and α-pinene, limonene, camphene, camphor, and myrcene [10]–[12]. Importantly, beyond their intrinsic antimicrobial activity, rosemary EOs have also been shown to enhance the efficacy of conventional antibiotics through synergistic interactions. Several studies reported that rosemary EO can potentiate the activity of many antimicrobial agents, including some aminoglycosides, tetracyclines, amphenicols, fluoroquinolone, and cephalosporins against Gram-negative pathogens, mainly through disruption of bacterial membranes and interference with efflux pump activity [13],[14]. However, like many medicinal and aromatic plants, the yield and chemical composition of rosemary EOs are strongly affected by various factors. These include the phenological stages of the plant, season of harvesting, bioclimatic conditions, geographical origin, organs, plant age among others [15]–[18]. Each of these elements can significantly influence the concentrations and types of bioactive compounds present in the oil, thus impacting its overall properties and efficacy. To optimize oil yields, specific chemical compositions, and enhance the biological activity of rosemary EO, research efforts have been focused on determining the ideal harvesting season under defined climatic conditions. Several studies indicate that the flowering period is crucial for harvesting rosemary's aerial parts for optimal EO extraction [19]–[23]. During this stage, rosemary significantly increases its production of secondary metabolites, a reaction possibly linked to enhanced pollinator activity [18]. This accumulation maximizes the richness of EOs in bioactive compounds, enhancing their therapeutic potential. In addition, cultivation under controlled aridity has been shown to increase the concentration and quality of rosemary EOs, making it a potentially interesting crop for marginal arid lands, particularly in Mediterranean regions [24]. However, it is well recognized that rosemary exhibits a prolonged flowering period, which complicates the determination of the optimal harvesting time for its EO, corresponding to the highest concentration of antimicrobial active compounds [25]. Given this extended flowering period of rosemary, this study aimed to (i) assess the monthly variation in EO yield and chemical profile throughout the flowering phase, (ii) evaluate monthly antimicrobial activity and synergistic interaction of rosemary EO with two conventional antibiotics (streptomycin and amphotericin B), and (iii) identify key compositional constituents influencing the observed bioactivity.

## 2. Materials and methods

### 2.1. Crop experimental design

This research was conducted on an experimental parcel situated in Oulad Dlim, Marrakech-Morocco (32°01′23″N/8°13′36″W, 388 m above sea level). The site is in a region with an arid bioclimate, with a mean maximum temperature of 38.3 °C in July and a mean minimum temperature of 4.5 °C in February, along with an average annual rainfall of 242 mm. The soil of experimental plot was a red clay loam type having a pH of 8.04, electrical conductivity of 0.49 mS/cm, organic matter content of 4.1%, total nitrogen at 0.66%, P_2_O_5_ at 18.20 mg/100 g, K_2_O at 49.9 mg/100 g, and CaCO_3_ at 34.1%. In March of 2018, during the vegetative development stage, 600 stem cuttings of rosemary were randomly selected from a natural population located in the Er-Rich region, Central of Morocco (32°08′25″N, 5°16′48″W, 1460 m above sea level). Clonal propagation was used to preserve the genetic identity of the natural population (chemotype). The cuttings were immediately transplanted into the Polyethylene bags (15/10) containing sand (1/3) and peat (2/3). After 4 weeks, rooted cuttings (15 to 20 cm high) were transplanted randomly to the experimental area with a planting distance of 0.5 m × 0.5 m. For the first month (establishment phase), a watering level equivalent to 60% of soil field capacity was provided early in the morning twice a week. For the next months, the young shoots were drip-irrigated weekly during the non-rainy season (dry season), with water applied at a rate of 5 liters per hour for three hours per session. No phytosanitary treatments were applied. In the period from September 2020 to March 2021, which corresponds to the flowering season, 20 flowering branches were randomly collected each month from a rosemary cultivated population. Monthly rainfalls and average monthly temperature, humidity and evaporation during the period of sample collection are reported in Table 1. Voucher specimens of cultivated (ROC-14) and wild (ROW-34) rosemary are deposited at the Laboratory of Microbial Biotechnology, Agrosciences and Environment, Faculty of Sciences, Cadi Ayyad University. Before EO extraction, the sampled plant materials (flowering branches) were air-dried in the shade at a temperature of 25 °C until reaching a constant weigh.

**Table 1.** Monthly rainfalls and average monthly temperature, humidity, and evaporation during the period of sample collection.

	Evaporation (mm)	Rainfalls (mm)	Temperature (°C)	Humidity (%)
SEP 2021	41.7	0.2	25.19	52.38
OCT 2021	31.8	0	22.73	46.25
NOV 2021	20.9	22	24.72	49.81
DEC 2021	17.4	32	14.28	60.44
JAN 2022	17.4	10.8	13.06	51.19
FEB 2022	19	35	16.07	52.06
MAR 2022	16.7	61	14.01	67

### 2.2. EO extraction and gas chromatography/mass spectrometry (GC/MS) analyses

EOs were extracted using a hydrodistillation process with a Clevenger-type apparatus (Borosil 3451029 Clevenger Apparatus) over a duration of 3 hours to ensure complete recovery. The process was conducted in triplicate (200 g × 3). The yields were determined by calculating the ratio of the volume of oil obtained to the weight of the dried plants used (v/w). The extracted EOs were then separated, dried using anhydrous sodium sulfate, and stored in opaque flasks at 4 °C in a dark environment until they were analyzed.

The qualitative and quantitative identification of the EO constituents was conducted using gas chromatography coupled to mass spectrometry (GC-MS) as previously described by Soulaimani et al. [26]. To accurately determine the compounds in each EO chromatographic profile during a single analysis, mass spectra were compared to known reference compounds when available, and consulted against the NBS75K and WILEY275 libraries, as well as a documented terpene library (Adams, 2007). Retention indices (RIs) were calculated based on the retention times of a range of C7–C30 n-alkanes using linear interpolation. These indices were then compared with those of known compounds or corroborated against published data to ensure accuracy.

### 2.3. Assessment of antimicrobial activity

The antimicrobial efficacy of the EOs was evaluated against four clinically isolated pathogenic Candida strains: *C. albicans* (CCMM L4), *C. glabrata* (CCMM L7), *C. krusei* (CCMM L10), and *C. parapsilosis* (CCMM L18). Additionally, three well-known pathogenic bacteria were tested: *Staphylococcus aureus* (CCMM B3), *Escherichia coli* (ATCC 8739), and *Salmonella enterica* (ATCC 14028). The antimicrobial activity of EOs was assessed using the agar disc diffusion and microwell dilution methods, in accordance with the Clinical and Laboratory Standards Institute (CLSI) guidelines [27]. For the disc diffusion assay, sterile discs (6 mm in diameter) impregnated with 10 µL of EOs were placed on the surface of either Sabouraud Dextrose Agar (SDA) or Mueller Hinton Agar (MHA) plates that had been previously inoculated with 0.1 mL of yeast or bacterial suspensions at concentrations of 10^5^ and 10^8^ CFU/mL, respectively. The plates were stored at 4 °C for four hours to facilitate EO diffusion, then incubated at 37 °C for 24 hours for bacterial cultures and at 28 °C for 48 hours for yeast cultures. Antimicrobial activity was determined by measuring the inhibition zones (IZ) around the discs. Streptomycin (15 µg/disc) and amphotericin B (5 µg/disc) served as positive controls, and all tests were conducted in triplicate.

In the microwell dilution method, EOs were diluted in a 2-fold series in 4% dimethyl sulfoxide (DMSO), with 100 µL of each dilution added to microwells that contained 100 µL of yeast or bacterial suspensions at densities of 1–2 × 10^3^ and 10^8^ CFU/mL, respectively. The microplates were incubated at 28 °C for 18–24 hours for *Candida* strains and at 37 °C for bacteria. The minimum inhibitory concentration (MIC) was defined as the lowest concentration of EOs that prevented the visible growth of the tested strains. To determine the minimum microbicidal concentration (MMC), 0.1 mL from each clear well, indicating no growth in MIC tests, was sub-cultured on appropriate agar (MHA or SDA) and incubated under the same conditions previously described. The MMC was identified as the lowest EO concentration that effectively killed 99,9% of the bacteria or yeasts. Streptomycin and amphotericin B were employed as standard antibacterial and antifungal agents, respectively.

### 2.4. Synergistic effect of EOs with standard antimicrobials

The interaction between rosemary EOs and two standard antimicrobial agents, amphotericin B for *Candida* spp. and streptomycin for bacteria, were evaluated using a checkerboard assay based on the broth microdilution method. Briefly, mixtures were prepared by combining 50 µL of the EO dilution with 50 µL of the antimicrobial agent dilution. Each well was then inoculated with 100 µL of microbial suspension. The microplates were incubated at 37 °C for 24 hours for bacterial strains and at 28 °C for 48 hours for *Candida* spp. The antimicrobial efficacy of each combination was assessed by calculating the MIC gain, which reflects the fold reduction in the MIC of the antimicrobial when used in combination. It was calculated using the formula: MIC gain = MIC of the antimicrobial alone/MIC of the antimicrobial in combination. To determine the nature of the interaction, the fractional inhibitory concentration index (FICI) was also calculated as follows: FICI = FIC (EO) + FIC (antimicrobial), where FIC (EO) = MIC of EO in combination/MIC of EO alone, and FIC (antimicrobial) = MIC of antimicrobial in combination/MIC of antimicrobial alone. All tests were conducted in triplicate. According to EUCAST standards, interactions were classified as synergistic when FICI ≤ 0.5, additive when 0.5 < FICI ≤ 1, indifferent when 1 < FICI ≤ 2, and antagonistic when FICI > 2 [28].

### 2.5. Statistical analysis

Data were expressed as the mean ± standard deviation, group means were compared by one way ANOVA and Tukey test to identify significance (p < 0.05) among groups using SPSS 21.0 statistical software.

## 3. Results and discussion

### 3.1. Yields and chemical compositions of rosemary EOs

**Figure 1.**
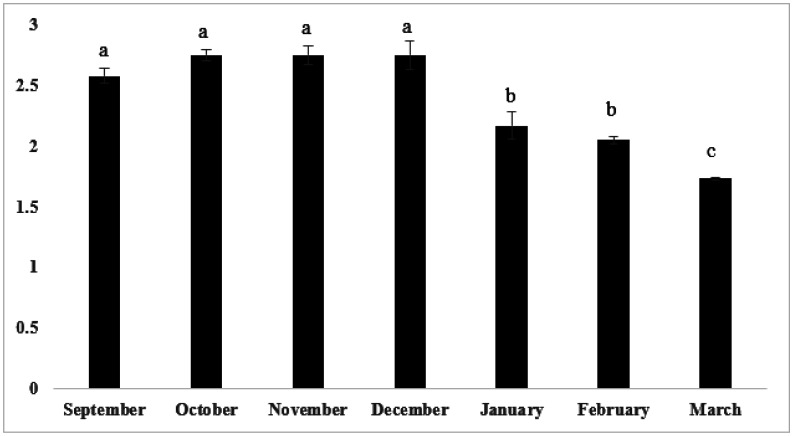
Monthly variation of rosemary EO yields during the flowering period.

The yields of EOs from rosemary, quantified as the volume of oil per weight of dried plant material, ranged between 1.73% and 2.75%. These values are comparatively higher than those reported for some rosemary populations cultivated under Mediterranean climate [25],[29]. Significant variations in EO yields were noted, with the highest yields occurring during the months of October, November, and December. In contrast, March, coinciding with the end of the flowering season, consistently showed the lowest EO productions (Figure 1). This trend is consistent with previous findings in both wild and cultivated rosemary, where higher EO yields were recorded during the autumn months, likely associated with climatic water stress conditions that favor the accumulation of secondary metabolites [19],[23],[25],[29],[30]. In fact, at our experimental site, this flowering season is characterized by reduced rainfall, elevated temperatures, and increased evapotranspiration rates, as shown in Table 1. The chemical composition of rosemary EOs collected throughout the flowering season (September to May) is presented in Table 2. The constituents, listed in order of elution, were identified using GC/MS analysis combined with retention indices. A total of 31 compounds were identified, representing between 97.69 ± 0.69% and 99.43 ± 0.03% of the total oil content. Monoterpenes constituted the predominant class across all EO samples. Sesquiterpenes, both hydrocarbon and oxygenated forms, were consistently present in low concentrations, confirming their scarcity in Moroccan Rosemary EOs, as reported in numerous previous studies [31],[32]. Regarding the main constituents of EOs, 1,8-cineole consistently exhibited the highest concentrations throughout the entire sampling period, ranging from 27.57 ± 0.76% to 35.30 ± 0.12% (Table 2). The other main compounds of EOs were α-pinene (15.36 ± 0.23%–28.97 ± 0.10%), camphor (7.12 ± 0.00%–15.37 ± 0.12%), β-pinene (6.21 ± 0.00%–10.24 ± 0.09%), borneol (3.26 ± 0.05%–5.68 ± 0.04%), and caryophyllene (4.36 ± 0.14–8.45 ± 0.05%). This result confirms the predominance of the 1,8-cineole/α-pinene/camphor chemotype in Moroccan rosemary, in line with findings from several previous studies [31]–[33]. The variation in the main EO components throughout the sampling period revealed that 1,8-cineole maintained the highest concentrations across all months, except in January, where it exhibited a marked decrease. Camphor was more prominent during autumn, followed by a gradual decline through winter and spring. In contrast, α-pinene exhibited significant monthly variation, reaching its highest concentration in March, which coincided with the end of the flowering period. This monthly variation is consistent with previous findings on rosemary EOs, which highlight the sensitivity of their main chemical constituents, particularly monoterpenes like 1,8-cineole, camphor, and α-pinene, to climatic factors [19],[31],[32],[34]. It is evident that these environmental influences can alter the biosynthesis and relative abundance of EO compounds, ultimately affecting the oil's biological activity.

**Table 2.** Chemical composition of rosemary EO during flowering period.

RT^b^	RI^c^	RI^lit^	Compounds^a^	SEP	OCT	NOV	DEC	JAN	FEB	MAR
2.63	924	924	α-Thujene	0.22 ± 0.15			0.39 ± 0.00	0.48 ± 0.00	0.39 ± 0.01	
2.71	936	932	α-Pinene	19.92 ± 0.96	18.96 ± 0.35	16.03 ± 0.18	15.85 ± 0.23	20.44 ± 0.08	15.36 ± 0.23	28.97 ± 0.10
2.85	945	946	Camphene	5.60 ± 0.12	4.81 ± 0.11	4.58 ± 0.09	5.05 ± 0.09	6.00 ± 0.07	5.60 ± 0.09	5.91 ± 0.05
2.96	977	974	1-Octen-3-ol	0.15 ± 0.05	0.14 ± 0.03	0.12 ± 0.00				
3.08	978	974	β-Pinene	6.74 ± 0.00	6.21 ± 0.00	6.29 ± 0.05	10.64 ± 0.08	9.29 ± 0.02	10.24 ± 0.09	8.49 ± 0.04
3.30	998	1002	α-Phellandrene	0.24 ± 0.00	0.29 ± 0.00	0.24 ± 0.00	0.21 ± 0.00	0.25 ± 0.00	0.19 ± 0.00	0.27 ± 0.00
3.42	1012	1014	α-Terpinene	0.70 ± 0.01	0.78 ± 0.02	0.60 ± 0.01	0.60 ± 0.02	0.72 ± 0.03	0.56 ± 0.02	0.73 ± 0.03
3.50	1025	1020	p-Cymene		0.40 ± 0.54	1.55 ± 0.02			1.26 ± 0.01	1.93 ± 0.01
3.61	1034	1026	1,8-Cineole	34.06 ± 0.50	33.96 ± 0.25	36.28 ± 0.26	34.94 ± 0.00	27.57 ± 0.76	35.30 ± 0.12	29.57 ± 0.09
3.90	1048	1054	γ-Terpinene	1.17 ± 0.04	1.18 ± 0.03	0.96 ± 0.02	1.15 ± 0.02	1.40 ± 0.02	1.13 ± 0.02	1.23 ± 0.01
4.03	1056	1065	Cis-Sabinene hydrate	0.11 ± 0.00	0.09 ± 0.00	0.09 ± 0.00	0.20 ± 0.00	0.09 ± 0.00	0.13 ± 0.00	0.05 ± 0.01
4.32	1082	1086	Terpinolene	0.55 ± 0.02	0.55 ± 0.00	0.44 ± 0.00	0.41 ± 0.00	0.63 ± 0.02	0.44 ± 0.00	0.64 ± 0.01
4.38	1098	1095	Linalool	0.49 ± 0.03	0.50 ± 0.03	0.43 ± 0.03	0.34 ± 0.02	0.44 ± 0.01	0.33 ± 0.01	0.65 ± 0.00
4.48	1105	1114	Fenchol	0.04 ± 0.00	0.06 ± 0.00	0.09 ± 0.00	0.05 ± 0.00	0.06 ± 0.00	0.06 ± 0.00	0.07 ± 0.00
4.75	1114	1124	Chrysanthenone	0.15 ± 0.00	0.15 ± 0.00	0.08 ± 0.00	0.09 ± 0.00	0.23 ± 0.01	0.11 ± 0.00	0.23 ± 0.00
5.30	1131	1141	Camphor	14.21 ± 0.05	14.51 ± 0.00	15.37 ± 0.12	13.48 ± 0.16	10.21 ± 0.06	14.19 ± 0.16	7.12 ± 0.00
5.65	1167	1165	Borneol	4.75 ± 0.15	4.43 ± 0.00	4.57 ± 0.01	4.63 ± 0.05	4.78 ± 0.00	5.68 ± 0.04	3.26 ± 0.05
5.84	1176	1174	Terpinen-4-ol	0.68 ± 0.02	0.80 ± 0.03	0.77 ± 0.04	0.69 ± 0.04	0.62 ± 0.02	0.73 ± 0.04	0.55 ± 0.02
6.08	1187	1186	α-Terpineol	2.12 ± 0.06	2.73 ± 0.09	2.61 ± 0.10	2.25 ± 0.13	1.91 ± 0.07	2.09 ± 0.08	1.52 ± 0.05
6.51	1204	1204	l-Verbenone	0.35 ± 0.00	0.39 ± 0.01	0.11 ± 0.00	0.07 ± 0.00	0.20 ± 0.00	0.05 ± 0.00	0.55 ± 0.01
8.11	1287	1284	Bornyl acetate	1.62 ± 0.01	1.52 ± 0.02	0.83 ± 0.01	1.07 ± 0.01	1.91 ± 0.01	1.28 ± 0.00	1.06 ± 0.00
8.44	1297	1298	Carvacrol	0.02 ± 0.01	0.05 ± 0.02			0.09 ± 0.01		
10.41	1376	1374	α-Copaene		0.03 ± 0.00			0.06 ± 0.00	0.01 ± 0.00	0.14 ± 0.00
11.60	1417	1417	Caryophyllene	4.36 ± 0.14	5.42 ± 0.07	6.31 ± 0.10	6.18 ± 0.01	8.45 ± 0.05	3.37 ± 0.00	4.28 ± 0.00
12.49	1453	1452	Humulene	0.60 ± 0.01	0.89 ± 0.00	0.76 ± 0.00	0.84 ± 0.01	1.22 ± 0.00	0.42 ± 0.00	0.94 ± 0.00
13.06	1477	1478	γ-Muurolene	0.03 ± 0.00			0.01 ± 0.00	0.05 ± 0.00		0.12 ± 0.00
13.63	1500	1500	Bicylogermacrene						0.11 ± 0.00	
13.97	1508	1505	β-Bisabolene	0.06 ± 0.01	0.09 ± 0.00	0.06 ± 0.00	0.02 ± 0.02	0.08 ± 0.00	0.02 ± 0.00	0.03 ± 0.00
14.10	1515	1513	γ-Cadinene	0.04 ± 0.00	0.04 ± 0.00		0.02 ± 0.00	0.05 ± 0.00	0.02 ± 0.00	0.11 ± 0.02
14.33	1524	1522	δ-Cadinene	0.07 ± 0.03	0.06 ± 0.00		0.01 ± 0.00	0.12 ± 0.00	0.01 ± 0.01	0.25 ± 0.00
16.00	1561	1582	Caryophyllene oxide	0.09 ± 0.01	0.13 ± 0.01	0.16 ± 0.00	0.20 ± 0.00	0.46 ± 0.00	0.27 ± 0.00	0.27 ± 0.00
			Oxygen-containing monoterpenes	57.00 ± 0.52	57.68 ± 0.08	60.41 ± 0.53	56.8 ± 0.42	46.10 ± 0.93	58.68 ± 0.46	43.56 ± 0.24
			Monoterpene hydrocarbons	35.15 ± 0.43	33.18 ± 0.03	30.68 ± 0.39	34.29 ± 0.49	39.19 ± 0.20	35.17 ± 0.49	48.17 ± 0.24
			Oxygen-containing sesquiterpenes	0.09 ± 0.01	0.13 ± 0.01	0.16 ± 0.00	0.20 ± 0.00	0.46 ± 0.00	0.27 ± 0.00	0.27 ± 0.00
			Sesquiterpene hydrocarbons	5.15 ± 0.19	6.53 ± 0.07	7.13 ± 0.10	7.07 ± 0.04	10.03 ± 0.06	3.95 ± 0.00	5.88 ± 0.02
			Other	1.76 ± 0.06	1.66 ± 0.01	0.95 ± 0.01	1.07 ± 0.01	1.91 ± 0.01	1.28 ± 0.00	1.06 ± 0.00
			Total	99.16 ± 0.17	99.18 ± 0.02	99.33 ± 0.03	99.43 ± 0.03	97.69 ± 0.69	99.36 ± 0.02	98.94 ± 0.02

^a^Compounds listed in order of elution; ^b^RT: Retention time; RI^c^: Retention indices relative to n-alkanes (C7–C30) on the TG-5MS capillary column; RI^lit^ : Retention indices from literature, according to Adams [35].

### 3.2. Antimicrobial assessment of the studied rosemary EOs

This part of our study aimed to assess whether monthly variations during the flowering period influence the antimicrobial activity of rosemary EOs. The antimicrobial properties were tested against seven clinically relevant pathogens: Four Candida species (*C. albicans*, *C. glabrata*, *C. parapsilosis*, *C. krusei*), two Gram-negative bacteria (*S. enterica* and *E. coli*), and one Gram-positive strain (*S. aureus*). Antimicrobial efficacy was determined through the evaluation of IZ, MIC, and MMC, with detailed results presented in Tables 3 and 4.

**Table 3.** Inhibition zone diameters (IZ in mm) determined by disc diffusion method for rosemary EOs collected during flowering season and Antibiotic.

	Essential oils							Antibiotics	
Microorganisms	SEP	OCT	NOV	DEC	JAN	FEV	MAR	Amphotericin B	Streptomycin
*C. glabrata* L7	9.88 ± 0.06^a^	10.91 ± 1.51	10.41 ± 1.69	8.74 ± 0.34	9.2 ± 0.52	6.52 ± 0.40	8.41 ± 0.84	11.5 ± 0.93	
C. *krusei* L10	13.31 ± 1.58	15.68 ± 1.24	16.49 ± 1.78	11.25 ± 1.34	10.72 ± 1.33	8.06 ± 0.32	13.25 ± 0.68	13.4 ± 0.62	
*C. parapsilosis* L18	11.47 ± 0.34	15.72 ± 0.73	14.3 ± 0.04	10.38 ± 0.2	8.94 ± 0.9	7.5 ± 0.55	10.4 ± 0.52	8.98 ± 1.15	
*C. albicans* L4	13.8 ± 1.22	15.07 ± 2.52	15.83 ± 0.39	12.2 ± 0.73	9.44 ± 1.26	7.73 ± 1.19	9.16 ± 0.41	10.56 ± 0.4	
*E. coli*	9.3 ± 0.05	9.6 ± 0.05	10.6 ± 0.05	9 ± 0.1	8.6 ± 0.2	9.6 ± 0.37	9 ± 0.1		14.8 ± 0.34
*S. aureus*	10 ± 0.35	11.3 ± 0.35	11.3 ± 0.05	10 ± 0.17	9.3 ± 0.05	8.6 ± 0.2	9.3 ± 0.05		17.7 ± 1.6
*S. enterica*	8.1 ± 0.25	10.1 ± 0.05	9.3 ± 0.12	9.6 ± 0.03	8.2 ± 0.2	8.1 ± 0.1	7.1 ± 0.21		13.08 ± 0.01

^a^Inhibition zone diameters include a disc diameter of 6 mm with a concentration of 10 µL of oil per disc, 15 µg of Streptomycin per disc, and 5 µg of amphotericin B per disc).

**Table 4.** MIC and MMC of rosemary EOs collected during the flowering season and standard antibiotics.

	Microorganisms tested													
	*C. glabrata* L7		*C. krusei* L10		*C. parapsilosis* L18		*C. albicans* L4		*E. coli*		*S. aureus*		*S. enterica*	
EOs/antibiotics	MIC	MMC	MIC	MMC	MIC	MMC	MIC	MMC	MIC	MMC	MIC	MMC	MIC	MMC
SEP	5	5	2.5	2.5	10	10	5	5	11.12	11.12	5.56	11.12	22.25	22.25
OCT	2.5	2.5	2.5	2.5	5	5	2.5	2.5	11.12	11.12	2.78	5.56	11.12	11.12
NOV	2.5	2.5	2.5	2.5	5	5	2.5	2.5	11.12	11.12	2.78	2.78	11.12	11.12
DEC	5	5	2.5	2.5	5	5	2.5	2.5	22.25	22.25	2.78	2.78	22.25	22.25
JAN	10	10	5	5	10	10	5	5	22.25	22.25	5.56	22.25	22.25	22.25
FEB	10	10	5	5	10	10	20	20	22.25	22.25	5.56	11.2	22.25	22.25
MAR	5	5	5	5	10	10	5	5	22.25	22.25	22.25	22.25	44.5	44.5
Amphotericin B	1.953	1.953	0.976	0.976	1.953	1.953	0.976	0.976						
Streptomycin									0.072	0.072	0.009	0.018	0.072	0.072

MIC and MMC are in mg/mL.

All rosemary EO samples collected monthly exhibited antimicrobial activity, with inhibition zone diameters revealing a more pronounced effect against fungal strains compared to bacterial ones (Table 3). For bacterial strains, the EO showed moderate activity. The strongest inhibition was observed against *S. aureus* in November (11.3 ± 0.05 mm), while the activity against *E. coli* and *S. enterica* was generally lower, with inhibition zones mostly below 10 mm. The highest recorded zone for *S. enterica* was in October (10.1 ± 0.05 mm). Among the yeasts, *C. krusei* showed the highest sensitivity, with inhibition zones reaching up to 16.49 ± 1.78 mm in November. *C. albicans* also exhibited strong sensitivity in November (15.83 ± 0.39 mm), whereas *C. glabrata* showed relatively lower inhibition values, with a minimum of 6.52 ± 0.40 mm in February. Regarding *C. parapsilosis*, the activity was highest in October and November (15.72 ± 0.73 mm and 14.3 ± 0.04 mm, respectively) and decreased during the later months. This higher susceptibility of fungi may be attributed to the greater permeability of fungal membranes to lipophilic constituents found in rosemary EOs, such as 1,8-cineole, camphor, and α-pinene, which are known to disrupt membrane integrity and impair cellular respiration [19],[36],[37]. The MIC values (Table 4) further supported the inhibitory potential of rosemary EOs, showing significant variation depending on the month of harvesting. The strongest antifungal activity was observed in oils collected in October and November, with MICs as low as 2.5 mg/mL across all *Candida* species. In contrast, weaker antifungal effects were recorded in January and February, with MICs rising to 10 or even 20 mg/mL, particularly against *C. albicans*. Regarding bacterial strains, *S. aureus* exhibited greater sensitivity to rosemary EOs, notably those from October and November (MIC: 2.78 mg/mL), whereas the Gram-negative strains *E. coli* and *S. enterica* were less susceptible, with MICs ranging from 11.12 to 44.5 mg/mL. This lower sensitivity is consistent with the structural features of Gram-negative bacteria, whose outer membrane acts as a barrier to hydrophobic compounds [38].

A clear seasonal trend in the antimicrobial efficacy of rosemary EOs emerges from these results. The observed month-to-month variation in bioactivity can be largely attributed to seasonal shift in the composition of major EO constituents, which are strongly influenced by climatic conditions. Notably, in October and November, the EOs showed enhanced antimicrobial and antifungal activities, likely associated with elevated concentrations of 1,8-cineole, camphor, and α-pinene, monoterpenes with well-documented antimicrobial properties [36],[39],[40]. These months were characterized by relatively low rainfall and high evaporation rates (Table 1), indicative of moderate climatic water stress (Table 1). Such stress conditions are known to induce adaptive metabolic responses in aromatic plants, including the upregulation of monoterpene biosynthesis pathways, particularly those leading to the accumulation of oxygenated compounds like 1,8-cineole and camphor[30]. In contrast, the reduced concentrations of these bioactive monoterpenes in the winter months (cooler climatic conditions) may account for the weaker inhibitory effects, particularly against *C. albicans* and Gram-negative bacteria. Nevertheless, as highlighted by Zaouali et al. (2010), the antimicrobial activity of rosemary EO is not solely attributable to its major compounds, but rather to the synergistic interactions between both major and minor constituents [20].

### 3.3. Synergistic antimicrobial effects of rosemary EO with antibiotics

A month-by-month analysis of the checkerboard assay results reveals clear seasonal trends in the synergistic activity of rosemary EOs when combined with streptomycin across all tested bacterial strains (Table 5). In *E. coli*, the strongest synergistic effect was observed during the early autumn months (September to November), with a consistent 32-fold reduction in the MIC of streptomycin and a FICI of 0.28, reflecting robust potentiation of antibiotic efficacy. This pronounced synergy likely correlates with the peak seasonal accumulation of oxygenated monoterpenes, in particular 1,8-cineole and camphor in the EOs, which are known to disrupt bacterial membranes and inhibit efflux pump activity [39],[40]. This membrane disruption likely enhances streptomycin penetration, facilitating its bactericidal effect and increasing bacterial mortality [41]. This mechanism is supported by Seukep et al. who demonstrated that plant-derived products can potentiate aminoglycoside activity against multidrug-resistant bacteria by interfering with efflux pump function [42]. From December to March, a notable decline in synergy was observed in *E. coli*, as the MIC gain dropped to 16-fold and the FICI slightly increased to 0.31, likely due to seasonal changes in the EO profile marked by a reduction in oxygenated monoterpenes. In March, despite an increase in α-pinene content (a hydrocarbon monoterpene), the synergistic effect remained unchanged (16-fold gain; FICI = 0.31), reinforcing the hypothesis that oxygenated monoterpenes play a more critical role in synergy than hydrocarbons alone [43]. In *S. aureus*, a Gram-positive strain, synergistic effects were also observed throughout all months, but with slightly lower MIC gains compared to *E. coli*. This difference may be attributed to structural differences in their cell envelopes, as the outer membrane of Gram-negative *E. coli* presents a stronger permeability barrier that is more markedly disrupted by EOs, thereby enhancing antibiotic uptake and synergy [44]. From September to December, the MIC of streptomycin was consistently reduced 8-fold, with FICI values of 0.37. Notably, the lowest EO MIC (0.695 mg/mL) was recorded in October and November, reflecting the increased potency of the EO during the autumn harvest. In January and February, the gain dropped to 4-fold, likely due to a moderate increase in ATB MIC and a potential decrease in EO membrane-disrupting compounds. Interestingly, a marked increase in EO MIC was observed in March (5.56 mg/mL), yet the MIC of streptomycin was strongly reduced (to 0.00056 mg/mL), yielding a 16-fold gain and a FICI of 0.31. This suggests that while EO efficacy may decline in terms of direct inhibition (higher MIC), it may still synergize effectively when paired with antibiotics, possibly through mechanisms beyond simple membrane disruption, such as efflux pump inhibition. *S. enterica* showed a more variable synergy profile. In October and November, the MIC gain peaked at 8-fold (ATB reduced from 0.018 to 0.009 mg/mL), with a FICI of 0.37, consistent with the autumnal peak in EO activity. During the colder months (December and February), the synergy weakened (4-fold MIC gain; FICI = 0.50), while a moderate rebound was observed in March, with MIC gains returning to 8-fold and a FICI of 0.37. These fluctuations likely reflect both compositional changes in EO and possible strain-specific sensitivity to seasonal chemical variations.

Regarding *Candida* spp., the synergistic effect of rosemary EOs with amphotericin B was overall limited and clearly strain and season dependent (Table 6). Among all tested isolates, only *C. glabrata* exhibited a true synergistic interaction in September (FICI = 0.50), accompanied by a 4-fold gain in the antibiotic's efficacy. This suggests that the EO profile during early autumn likely richer in oxygenated monoterpenes (1,8-cineole and camphor) may potentiate the antifungal activity of amphotericin B. In contrast, from October to March, the interaction in *C. glabrata* consistently dropped to an additive level (FICI = 0.75), with only a 2-fold gain in MIC, indicating a seasonal loss of bioactive synergy. Similarly, in *C. albicans*, a temporary additive effect was observed in September and October, followed by a shift to indifference (FICI = 1.25) in the subsequent months. This decline in synergistic potential may reflect seasonal changes in EO composition, especially the reduction of compounds that facilitate membrane disruption or ergosterol accessibility. For *C. krusei*, the interaction remained additive throughout the entire flowering period (FICI = 0.75), with stable MIC values and no significant seasonal fluctuation, suggesting a moderate but consistent EO–drug interaction that was insufficient to reach synergy. The most resistant profile was observed in *C. parapsilosis*, where the interaction remained indifferent across all months (FICI = 1.25), with no gain in amphotericin B activity. The limited synergistic activity observed with amphotericin B, particularly in *C. parapsilosis*, and *C. albicans* interactions, may be attributed to the polyene mode of action of the antibiotic. Amphotericin B binds to ergosterol in fungal cell membranes to form pores, thereby increasing membrane permeability and causing cell death [45]. It appears that certain components of rosemary EO may intercalate into the fungal lipid bilayer, altering its structural integrity and membrane environment. This modification could inadvertently reduce amphotericin B's access to ergosterol or disrupt membrane fluidity in a manner that impairs the formation of transmembrane pores. Consequently, rather than potentiating the antifungal activity of amphotericin B, co-treatment with rosemary EO may partially antagonize its binding efficiency, thereby diminishing the expected synergistic effect as reported in previous studies [45]. Consequently, rather than potentiating the antifungal activity of amphotericin B, co-treatment with rosemary EO may partially antagonize its binding efficiency, thereby diminishing the expected synergistic effect.

**Table 5.** Fractional inhibitory concentration indices (FICI) and synergistic interactions between rosemary EOs collected during the flowering season and streptomycin against bacterial strains.

Bacterial strains		MICc		Gain	FIC		FICI	Interaction
		EO	ATB		EO	ATB		
*E. coli*	SEP	2.78	0.00225	32	0.25	0.03	0.28	Synergism
	OCT	2.78	0.00225	32	0.25	0.03	0.28	Synergism
	NOV	2.78	0.00225	32	0.25	0.03	0.28	Synergism
	DEC	5.56	0.0045	16	0.25	0.06	0.31	Synergism
	JAN	5.56	0.0045	16	0.25	0.06	0.31	Synergism
	FEB	5.56	0.0045	16	0.25	0.06	0.31	Synergism
	MAR	5.56	0.0045	16	0.25	0.06	0.31	Synergism
*S. aureus*	SEP	1.39	0.0011	8	0.25	0.12	0.37	Synergism
	OCT	0.695	0.0011	8	0.25	0.12	0.37	Synergism
	NOV	0.695	0.0011	8	0.25	0.12	0.37	Synergism
	DEC	0.695	0.0011	8	0.25	0.12	0.37	Synergism
	JAN	1.39	0.00225	4	0.25	0.12	0.37	Synergism
	FEB	1.39	0.00225	4	0.25	0.25	0.50	Synergism
	MAR	5.56	0.00056	16	0.25	0.06	0.31	Synergism
*S. enterica*	SEP	5.56	0.018	4	0.25	0.25	0.50	Synergism
	OCT	2.78	0.009	8	0.25	0.12	0.37	Synergism
	NOV	2.78	0.009	8	0.25	0.12	0.37	Synergism
	DEC	5.56	0.018	4	0.25	0.25	0.50	Synergism
	JAN	5.56	0.009	8	0.25	0.12	0.37	Synergism
	FEB	5.56	0.018	4	0.25	0.25	0.50	Synergism
	MAR	11.12	0.009	8	0.25	0.12	0.37	Synergism

MIC EO, MIC of EO in combination in mg/mL; MIC ATB, MIC of ATB in combination in mg/mL; FICI, fractional inhibitory concentration index.

**Table 6.** FICI and synergistic interactions between rosemary EOs collected during the flowering season and amphotericin B against candida species.

Candida *spp*.		MIC EO	MIC ATB	Gain	FIC EO	FIC ATB	FICI	Interaction
*C. glabrata L7*	SEP	1.25	0.488	4	0.25	0.25	0.50	Synergism
	OCT	0.625	0.976	2	0.25	0.50	0.75	Additive
	NOV	0.625	0.488	4	0.25	0.50	0.75	Additive
	DEC	1.25	0.976	2	0.25	0.50	0.75	Additive
	JAN	2.5	0.976	2	0.25	0.50	0.75	Additive
	FEB	2.5	0.976	2	0.25	0.50	0.75	Additive
	MAR	1.25	0.976	2	0.25	0.50	0.75	Additive
*C. krusei L10*	SEP	0.625	0.488	2	0.25	0.50	0.75	Additive
	OCT	0.625	0.488	2	0.25	0.50	0.75	Additive
	NOV	0.625	0.488	2	0.25	0.50	0.75	Additive
	DEC	0.625	0.488	2	0.25	0.50	0.75	Additive
	JAN	1.25	0.488	2	0.25	0.50	0.75	Additive
	FEB	1.25	0.488	2	0.25	0.50	0.75	Additive
	MAR	1.25	0.488	2	0.25	0.50	0.75	Additive
*C. parapsilosis L18*	SEP	2.5	1.953	1	0.25	1	1.25	Indifference
	OCT	1.25	1.953	1	0.25	1	1.25	Indifference
	NOV	1.25	1.953	1	0.25	1	1.25	Indifference
	DEC	1.25	1.953	1	0.25	1	1.25	Indifference
	JAN	2.5	1.953	1	0.25	1	1.25	Indifference
	FEB	2.5	1.953	1	0.25	1	1.25	Indifference
	MAR	2.5	1.953	1	0.25	1	1.25	Indifference
*C. albicans L4*	SEP	2.5	0.976	2	0.25	0.50	0.75	Additive
	OCT	0.625	0.488	2	0.25	0.50	0.75	Additive
	NOV	0.625	0.976	1	0.25	1	1.25	Indifference
	DEC	0.625	0.976	1	0.25	1	1.25	Indifference
	JAN	2.5	0.976	1	0.25	1	1.25	Indifference
	FEB	5	0.976	1	0.25	1	1.25	Indifference
	MAR	2.5	0.976	1	0.25	1	1.25	Indifference

MIC EO, MIC of EO in combination in mg/mL; MIC ATB, MIC of ATB in combination in mg/mL; FICI, fractional inhibitory concentration index.

## 4. Conclusions

This study provides compelling evidence that the antimicrobial potential of *R. officinalis* EOs is profoundly influenced by seasonal variation during the flowering period in arid Mediterranean conditions. The findings clearly demonstrate that EOs collected during autumn, particularly in October and November with relatively high climatic water stress, exhibit the highest yields and superior antimicrobial efficacy, especially against *Candida* species and the Gram-positive *S. aureus*. This enhanced bioactivity was closely associated with elevated levels of oxygenated monoterpenes, in particular 1,8-cineole and camphor. Importantly, the synergistic assays revealed that rosemary EOs markedly potentiated the antibacterial action of streptomycin, achieving up to a 32-fold MIC reduction against *E. coli* in autumn, suggesting a promising role in overcoming antibiotic resistance in this Gram-negative bacterium. However, this synergy was more modest in *S. aureus* and more variable in *S. enterica*, reflecting strain-specific differences EO susceptibility. In contrast, the co-administration of rosemary EOs with amphotericin B yielded limited effects against *Candida* spp. These findings suggests that rosemary EOs harvested in early autumn, enriched in oxygenated monoterpenes, may act as effective natural adjuvants to enhance the efficacy of antibiotics and potentially enabling dose reductions that minimize toxicity. Standardizing harvest time to early autumn could thus optimize the therapeutic value of rosemary EO formulations and support their integration into antimicrobial strategies targeting some resistant multidrug resistant bacteria, including the Gram-negative E. coli. To facilitate their clinical integration, further *in vivo* pharmacological evaluations, toxicological assessments, and well-designed clinical trials are necessary to validate their safety, bioavailability, and synergistic efficacy when co-administered with conventional antibiotics. Nevertheless, the partial antagonism observed with amphotericin B warrants caution and highlights the need for strain-specific and drug-specific compatibility assessments before clinical use. These interactions must be considered when designing combinatory therapies to avoid compromising drug efficacy.

## Use of AI tools declaration

The authors declare they have not used Artificial Intelligence (AI) tools in the creation of this article.
